# Vitamin D as a Novel Regulator of Tumor Metabolism: Insights on Potential Mechanisms and Implications for Anti-Cancer Therapy

**DOI:** 10.3390/ijms18102184

**Published:** 2017-10-19

**Authors:** Mohamed A. Abu el Maaty, Stefan Wölfl

**Affiliations:** Institute of Pharmacy and Molecular Biotechnology, University of Heidelberg, Im Neuenheimer Feld 364, 69120 Heidelberg, Germany; abu.el.maaty@gmail.com

**Keywords:** vitamin D, cancer, metabolism, autophagy, AMPK (AMP-activated protein kinase), mTOR (Mammalian target of rapamycin), TXNIP (Thioredoxin-interacting protein), p53, HIF1a (Hypoxia-inducible factor 1a), c-Myc

## Abstract

1,25-dihydroxyvitamin D_3_ [1,25(OH)_2_D_3_], the bioactive form of vitamin D, has been shown to possess significant anti-tumor potential. While most studies so far have focused on the ability of this molecule to influence the proliferation and apoptosis of cancer cells, more recent data indicate that 1,25(OH)_2_D_3_ also impacts energy utilization in tumor cells. In this article, we summarize and review the evidence that demonstrates the targeting of metabolic aberrations in cancers by 1,25(OH)_2_D_3_, and highlight potential mechanisms through which these effects may be executed. We shed light on the ability of this molecule to regulate metabolism-related tumor suppressors and oncogenes, energy- and nutrient-sensing pathways, as well as cell death and survival mechanisms such as autophagy.

## 1. Tumor Metabolism: The Newest Hallmark of Cancer

Normal cells efficiently break down glucose through multi-step processes, namely glycolysis, the tricarboxylic acid (TCA) cycle, and oxidative phosphorylation, to yield reducing equivalents and ATP [1]. These processes are influenced by a number of factors including the environment’s oxygenation. Under aerobic conditions, glucose metabolism takes place in both the cytoplasm and mitochondria to maximize ATP production. However, under anaerobic conditions, metabolism is shifted from mitochondrial respiration to glycolysis—an observation known as the “Pasteur effect”—which ends with the conversion of pyruvate to lactate to restore NAD+ levels [2]. Cancer cells on the other hand, exhibit enhanced glucose fermentation independent of the environment’s oxygenation [1]. This phenomenon was originally identified by Otto Warburg early in the twentieth century and was subsequently termed the “Warburg effect” or “aerobic glycolysis” [1]. Over the decades since the original discovery, several explanations for this perplexing phenomenon have been proposed; however, recent studies have shown that the metabolic alterations observed in cancers are the result of complex and possibly coordinated actions of mutated/amplified proto-oncogenes and tumor suppressors [3]. Moreover, the expression/activity of several “metabolic genes” have been shown to be altered in tumors, for example glucose transporter 1 (GLUT1) [4], pyruvate kinase M2 (PKM2) [5], as well as lactate dehydrogenase A (LDHA) [6], making them potential druggable targets.

The resurrection of this cancer hallmark has prompted investigations into diverse metabolism-related therapeutic options [7]. Several novel drugs have been developed to enhance the wild-type activity of relevant tumor suppressors, e.g., using nutlin-3a to amplify p53-signaling [8], or alternatively inhibit oncogenic signaling, e.g., using Myc transactivation inhibitors [9]. Furthermore, various molecules that influence glucose metabolism are currently being investigated as potential anti-cancer drugs, such as the glycolytic inhibitor 2-deoxyglucose and the mitochondrial complex I inhibitor metformin [7].

Besides aberrations in glucose metabolizing pathways, accumulating evidence has illustrated that cancer cells have an increased demand for different amino acids, most notably glutamine, known as the phenomenon of “glutamine addiction” [10]. These amino acids are required, for example, in TCA cycle anaplerosis, the conversion of glutamine to glutamic acid and then α-ketoglutarate by the enzymes glutaminase and glutamic acid dehydrogenase [10], respectively, as well as for the maintenance of the redox balance and anabolic processes, e.g., serine, through contribution to the folate cycle [11]. Furthermore, alterations in both fatty acid biosynthesis and beta-oxidation have been observed in cancers, and several drugs targeting key enzymes in both processes have demonstrated promising anti-cancer effects, for instance orlistat [12], the fatty acid synthase inhibitor, as well as perhexiline, the carnitine palmitoyltransferase I inhibitor [13].

In addition to the aforementioned drugs, accumulating evidence has pointed towards the ability of the hormonally active form of vitamin D, 1,25-dihydroxyvitamin D_3_ [1,25(OH)_2_D_3_] (also known as calcitriol), to influence energy utilization in cancer cells [14,15,16,17].

## 2. Anti-Cancer Effects of Vitamin D: Possible Regulation of Metabolic Networks

Vitamin D is a seco-steroid that is now known to possess activities beyond the maintenance of good skeletal health, such as the regulation of proliferation, metabolism, and immunomodulation [18]. With regards to cancer, large clinical trials are underway to assess the molecule’s therapeutic utility, whereas in vitro and in vivo studies have largely demonstrated profound anti-tumor potential associated with the active form [19,20]. Additionally, 1,25(OH)_2_D_3_ and its analogues have been shown to be promising candidates for combination chemotherapy [21], due to their ability to augment the effects of conventional anti-cancer drugs including various anti-metabolites and platinum-based drugs.

Among the well-characterized anti-tumor actions 1,25(OH)_2_D_3_ exerts is its ability to regulate the expression of an array of molecules that work to impede proliferation, such as p21 and p27, or induce/facilitate apoptosis, such as induction of the pro-apoptotic molecule BAX, and reduction of the anti-apoptotic molecule Bcl-2 [19]. Calcitriol has also been shown to decrease the expression of the oncogenes hypoxia-inducible factor 1a (HIF1a) and c-Myc [19]. Moreover, studies have demonstrated extensive crosstalk between the vitamin D receptor (VDR) and the tumor suppressor p53 [22]. These effects, in addition to other activities described extensively elsewhere [19,20], have been reported in various experimental models, and currently serve as the main body of evidence of the anti-cancer potential of 1,25(OH)_2_D_3_.

Interestingly, a number of direct and indirect cellular targets/interactors of calcitriol have been implicated in tumor metabolism, besides their primary roles in regulating survival, such as c-Myc [23], HIF1a [24], and p53 [25]. In the following sub-sections, we summarize the effects of 1,25(OH)_2_D_3_ on metabolism-related oncogenes and tumor suppressors, and postulate on the potential metabolic outcome of this regulation. We also highlight the results of recent studies demonstrating the ability of VDR activators to influence metabolic signaling molecules, namely AMP-activated protein kinase (AMPK) [26], mammalian target of rapamycin (mTOR) [27], and thioredoxin-interacting protein (TXNIP) [17].

### 2.1. Regulation of c-Myc and HIF1a by 1,25(OH)_2_D_3_ and Potential Impact on Metabolism

c-Myc and HIF1a are transcription factors that are implicated in tumor proliferation and survival. The former is estimated to be amplified in 70% of all human tumors, whereas levels of the latter are known to increase under hypoxic conditions, which occur when tumor cell proliferation surpasses the oxygenation capacity of its environment’s vascularization [28]. It is now known that the two moieties rewire tumor metabolism, acting on both similar and distinct targets, to tailor nutrient utilization for optimal survival [28]. For example, GLUT1 and LDHA have been shown to be induced by both transcription factors, whereas pyruvate dehydrogenase kinase (PDK) isozyme 1 and FASN (Fatty acid synthase) are regulated by HIF1a and c-Myc, respectively [28].

Reduction of the expression of both factors by 1,25(OH)_2_D_3_ in cancer cells has been reported by numerous studies [29,30,31], and the genes encoding these oncogenes have been shown to harbor putative vitamin D response elements (VDRE) [32,33]. Given the important roles these molecules play in regulating tumor metabolism [28], it is not surprising that in cells where they were found to be negatively regulated by 1,25(OH)_2_D_3_, metabolic reprogramming was also observed. For example, in the prostate cancer cell line LNCaP, 1,25(OH)_2_D_3_ treatment has been shown to reduce c-Myc expression and HIF1 transcriptional activity [29,30]. We have recently shown that in the same cell line, 1,25(OH)_2_D_3_ induces profound changes in glucose-metabolizing pathways, including a clear reduction in mRNA and protein expression of both GLUT1 and PDK isozyme 1, as well as LDHA mRNA levels and overall lactate production [17]. While we did not investigate the role of c-Myc and HIF1a in mediating these effects, it is possible that the observed metabolic phenotype is partly achieved through the effect of 1,25(OH)_2_D_3_ on them.

### 2.2. 1,25(OH)_2_D_3_ and p53: Commonalities and Diversities in Metabolic Regulation

Mutations in the *TP53* gene are frequently occurring in malignancies, and are implicated in the pathogenesis of different tumor types [34]. These mutations essentially impact the ability of p53 to execute its canonical response to genotoxic stress. In cells harboring “mutant p53”, a comprehensive survival program is triggered that confers resistance to chemotherapeutics and enhances cancer cells’ invasion, migration, and proliferation [34]. Recent work has demonstrated that besides controlling the cell cycle and apoptotic signaling, p53 acts as a powerful regulator of tumor metabolism [25]. As previously mentioned, profound crosstalk between p53 and the VDR has been reported in a number of contexts, where studies have shown that the *VDR* gene is a direct target of p53 and its family members [22]. Moreover, the presence of mutant p53 has been shown to influence the anti-cancer effects of 1,25(OH)_2_D_3_, converting it from a tumor suppressing agent into a pro-survival one [35]. Since p53’s newly recognized metabolic roles are highly versatile and impact major nutrient-metabolizing pathways [25], we focus here on metabolic targets of p53 that are also regulated by 1,25(OH)_2_D_3_ in either similar or opposing fashions.

The main effect of p53 on glucose metabolism is to hamper aerobic glycolysis and induce mitochondrial respiration [25]. Through reducing the expression of GLUT1 and 4, as well as that of monocarboxylase transporter 1 (lactate’s efflux transporter), p53 dampens overall glycolytic flux [25]. Additionally, p53 induces mitochondrial respiration through distinct mechanisms, including the reduction of PDK isozyme 2 expression—the enzyme responsible for phosphorylating and subsequently inhibiting the activity of the pyruvate dehydrogenase complex—thereby enhancing the conversion of pyruvate to acetyl-CoA and further entry into the TCA cycle [25]. Furthermore, with regards to TCA cycle regulation, Tsui et al. [36] illustrated that the induction of p53 levels in prostate cancer cells through camptothecin treatment or by an expression vector markedly reduced mitochondrial aconitase expression. Moreover, p53 has been shown to enhance glutamine-driven TCA cycle anaplerosis by inducing glutaminase 2 expression [25].

Regarding the effects of 1,25(OH)_2_D_3_ on glucose metabolism, studies have illustrated that the molecule influences glycolysis on different levels, including glucose uptake and lactate production [14,17]. For example, similar to p53, calcitriol has been shown to reduce GLUT1 expression in different prostate cancer cells [17,29]. On the other hand, recent reports have demonstrated the differential regulation of expression of PDK isozymes by 1,25(OH)_2_D_3_ in different cell types. For instance, PDK1 expression was found to be reduced by 1,25(OH)_2_D_3_ treatment in prostate cancer cells [17], but unaffected in H-ras transformed breast epithelial cells [14]. Furthermore, in human dendritic and skeletal muscle cells, 1,25(OH)_2_D_3_ treatment was shown to induce and reduce the mRNA expression of other PDK isozymes, PDK3 and PDK4, respectively [37,38]. Interestingly, Contractor and Harris demonstrated that the expression of PDK2, but not PDK1, is p53-dependent [39]. It is therefore possible that 1,25(OH)_2_D_3_ treatment and p53 induction may lead to a similar metabolic phenotype, independent of one another, e.g., an overall net effect of increased pyruvate to acetyl-coA conversion through the downregulation of different PDK isozymes. However, in scenarios where possible mediators of calcitriol’s metabolic effects (e.g., HIF1a) are also influenced by p53, certain metabolic targets may be similarly regulated.

A clear diverging point in metabolic regulation by 1,25(OH)_2_D_3_ and p53 is glucose-6-phosphate dehydrogenase (G6PD). This enzyme catalyzes the first committed step in the pentose phosphate pathway (PPP), and is the main source of cellular NADPH, which is required for anti-oxidant defense mechanisms [40]. Additionally, G6PD levels have been implicated in different pathologies, for example deficiency in hemolysis and overexpression in cancers [40].

Vitamin D is a known positive regulator of G6PD expression and activity [40,41,42,43]. In non-malignant prostate epithelial cells, the G6PD gene has been shown to harbor VDRE, and to be strongly induced by 1,25(OH)_2_D_3_ treatment, preventing oxidative damage-mediated cellular death [43]. It is noteworthy that, in the same study, the authors did not observe an induction in G6PD expression with treatment in malignant prostate cells [43]. On the other hand, Simmons et al. [42] illustrated that 1,25(OH)_2_D_3_ induces G6PD mRNA expression in both cancerous and non-cancerous mammary cells, which altogether highlights the ability of calcitriol to induce the expression of this enzyme in different tissues, which—in certain tissues—was found in both healthy and malignant cells.

While G6PD induction by 1,25(OH)_2_D_3_ is beneficial in pre-malignancy, and thus in chemoprevention, as demonstrated by Bao et al. [43], the induction of G6PD expression/activity by 1,25(OH)_2_D_3_ in cancer cells could possibly be pro-survival, since (i) G6PD is a putative oncogene that is overexpressed in many cancers [40], and (ii) due to the possible subsequent increase in the rate of the PPP, which could provide rapidly proliferating cells with precursors for anabolic processes [44]. However, it is possible that this induction either also contributes to the molecule’s anti-cancer effects, for example through increased cellular anti-oxidant defense, or is simply not detrimental to the otherwise overall tumor suppressing action of the molecule. In support of the latter are the potent and diverse anti-tumor effects of calcitriol shown to be induced in the breast cancer cell line MCF-7 [45,46], which also exhibited elevated G6PD expression in response to the treatment [42].

Regulation of the PPP by p53 appears to be contradictory, with both inducing and inhibiting roles described [25]. The degree of stress/p53 activation has been proposed to be a determinant of the nature of PPP regulation by p53 [25]. In contrast to the effect of 1,25(OH)_2_D_3_, p53 has been shown to bind to and inhibit G6PD [25]. On the other hand, the p53 target gene *TIGAR* (TP53-induced glycolysis and apoptosis regulator), acts as a bisphosphatase, reducing the levels of fructose-2,6-bisphosphate, which acts as a positive regulator of the rate-limiting glycolytic enzyme phosphofructokinase 1 (PFK1) [25]. In doing so, TIGAR reduces the glycolytic rate and increases the availability of intermediates upstream of PFK1 for shunting into the PPP [25]. Therefore, under certain conditions, p53 and 1,25(OH)_2_D_3_ may act to induce a similar metabolic phenotype—PPP induction—through different mechanisms. However, in instances where p53 signaling is highly activated, G6PD could be differentially regulated by p53 and 1,25(OH)_2_D_3_. It will therefore be interesting to investigate the possible outcome, in terms of G6PD regulation and anti-tumor effects, when combining 1,25(OH)_2_D_3_ with either a p53 activator such as nutlin-3a, or a G6PD inhibitor, e.g., dehydroepiandrosterone.

### 2.3. Regulation of the AMPK-mTOR-TXNIP Signaling Triad by 1,25(OH)_2_D_3_

In addition to the effect of 1,25(OH)_2_D_3_ on the aforementioned metabolism-related transcription factors, the molecule has been shown to regulate the activity of other signaling pathways implicated in nutrient utilization, such as the converging AMPK, mTOR, and TXNIP metabolic signaling triad.

AMPK is a heterotrimeric complex that consists of an α catalytic sub-unit, as well as β and γ regulatory sub-units [47]. It is a pivotal intracellular energy sensor that responds to energetic stress, i.e., an increase in AMP:ATP ratio, by inhibiting energy-consuming processes, such as fatty acid and cholesterol synthesis, and inducing energy-generating processes, including glucose uptake and fatty acid oxidation [47]. Under metabolic stress, AMP/ADP molecules bind to regulatory sub-units of the complex, facilitating the phosphorylation of threonine-172 of the α sub-unit by the upstream kinase liver kinase B1 (LKB1), which subsequently activates the enzyme [47]. Additionally, an increase in intracellular Ca^2+^ levels activates AMPK signaling, through inducing another upstream kinase—calcium-calmodulin dependent kinase kinase 2 (CAMKK2) [47]. Interestingly, a controversial role for AMPK signaling has been observed in cancers, with both tumor-suppressing and -promoting effects reported in the literature [48]. With regards to tumor survival, AMPK has been shown to be activated in prostate cancer human samples, and that inhibiting this pathway by small interfering RNA or by the small molecule compound C (an AMPK inhibitor) inhibits cellular proliferation [49]. Additionally, AMPK activation is known to induce autophagy [47], the role of which is also controversial in tumors [50]. Advocating AMPK’s role in tumor suppression is its activation by the well-characterized tumor suppressor LKB1 [48], the clear anti-cancer potential of AMPK activators (e.g., metformin) [47], and the ability of AMPK to inhibit pro-survival downstream targets, namely mTOR [51].

mTOR signaling regulates a variety of critical cellular processes including growth, proliferation, and metabolism [51]. In cancers, among other pathologies, this pathway appears to be deregulated, with drugs inhibiting it—for example rapalogues (rapamycin analogues)—being investigated as therapies against different tumor types [51]. In contrast to AMPK’s cellular effects, mTOR activation leads to an increase in anabolic processes such as protein and lipid synthesis, and inhibits catabolic processes, such as autophagy [51].

The third member of this metabolic triad, TXNIP, has been recently shown to be a component of AMPK signaling [52]. TXNIP was originally identified in HL-60 cells by Chen and DeLuca as the vitamin D_3_-upregulated protein 1 (VDUP1) [53]. Subsequent studies have shown that TXNIP binds to and negatively regulates thioredoxin function [54], hence its name. Additionally, the molecule has been shown to act as an intracellular glucose sensor, responding to increases in glycolytic intermediates by limiting glucose uptake [55]. A recent study demonstrated that AMPK activation leads to an induction in glucose uptake by inducing TXNIP degradation [52]. Furthermore, different studies have shown that the inhibition of mTOR induces TXNIP expression [56], and that TXNIP contributes to mTOR signaling inhibition [57].

**Figure 1:**
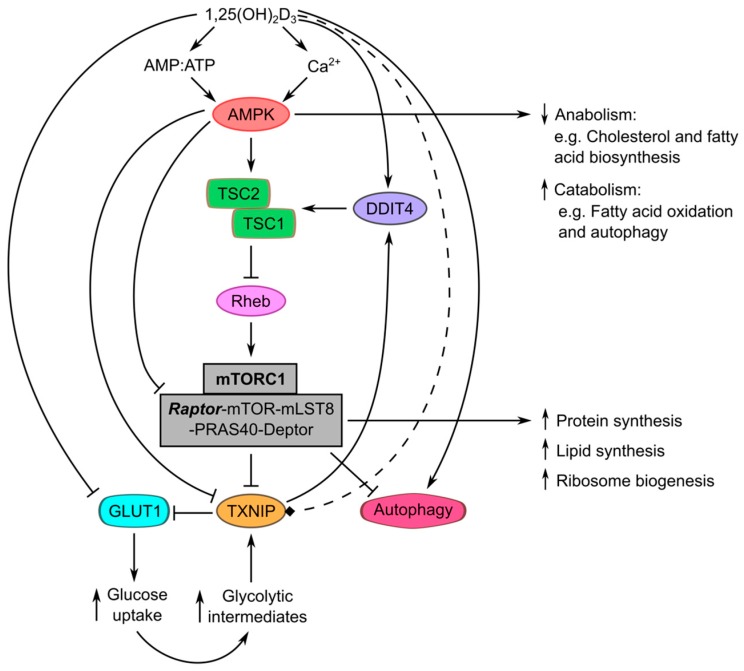
Multi-level regulation of the AMPK-mTOR-TXNIP signaling triad by 1,25(OH)_2_D_3_ and potential impact on metabolism. Induction of AMPK signaling by 1,25(OH)_2_D_3_, through increasing the AMP:ATP ratio or intracellular Ca^2+^ levels, may lead to differential TXNIP regulation. On one hand, AMPK directly phosphorylates TXNIP and marks it for degradation. On the other hand, AMPK activation may lead to an increase in TXNIP levels, through increasing the availability of the TXNIP-regulating transcriptional machinery, namely MondoA (not depicted in figure), as a result of mTOR signaling inhibition. mTOR inhibition secondary to AMPK activation is achieved by two mechanisms: (i) through phosphorylating TSC2 (tuberous sclerosis complex 2), which inhibits the activity of the mTOR stimulator Rheb (Ras homologue enriched in brain), and (ii) through phosphorylating Raptor (regulatory-associated protein of mTOR), thereby inhibiting mTOR complex 1 (mTORC1) activity. Additionally, calcitriol may also inhibit mTOR signaling through inducing the expression of DDIT4, which enables the assembly/activation of TSC1/2. This may lead to an increase in TXNIP levels, which in turn could inhibit mTOR signaling through stabilizing DDIT4. It is, however, unclear whether 1,25(OH)_2_D_3_ directly regulates TXNIP expression or not (hence this action is depicted using a dashed line). Furthermore, the induction of glucose uptake by AMPK activation conflicts with the demonstrated ability of 1,25(OH)_2_D_3_ to reduce the expression of GLUT1 in cancer cells. Moreover, the induction of autophagy by VDR activators has been shown to involve AMPK signaling, as well as the direct regulation of autophagy genes. We propose that calcitriol treatment regulates energy utilization of cancer cells through multiple mechanisms, including the regulation of the AMPK-mTOR-TXNIP triad. Arrows indicate induction and blunted arrows (T bar) indicate inhibition.

An increasing number of studies point towards the ability of 1,25(OH)_2_D_3_ and its analogues to regulate components of the AMPK-mTOR-TXNIP signaling triad (Figure 1) [17,26,27]. However, a clear connection to metabolic rewiring in tumor cells is yet to be fully characterized. Additionally, the distinct nature of regulation of individual components of this pathway by calcitriol may lead to intricate, seemingly non-canonical, and potentially counter-therapeutic outcomes. For instance, the 1,25(OH)_2_D_3_-mediated activation of AMPK through non-genomic or indirect effects, e.g., through increasing intracellular Ca^2+^ levels or the AMP:ATP ratio, may lead to the phosphorylation and subsequent degradation of TXNIP. This mechanism has been proposed in prostate cancer cells [17], and appears to be a therapeutic paradox since TXNIP is currently viewed as a promising tumor suppressor, due to its ability to induce apoptosis in cancer cells [58]. Furthermore, the negative regulation of glucose transporters by 1,25(OH)_2_D_3_, as shown in the case of GLUT1 in cancer cells [17,29], may reduce glucose uptake and intracellular levels of glycolytic intermediates capable of inducing TXNIP expression. On the other hand, the inhibition of mTOR signaling by 1,25(OH)_2_D_3_, e.g., as demonstrated by Lisse et al. [27] through inducing the expression of the negative regulator of mTOR, DDIT4 (DNA damage inducible transcript 4), also known as REDD1 (regulated in development and DNA damage response 1), may lead to an induction in TXNIP levels, which may reciprocally participate in mTOR signaling inhibition, as shown by Jin et al. [57]. Moreover, it is important to note that, despite its name, the *TXNIP/VDUP1* gene has not been shown to be directly regulated by the VDR, and that VDRE have not been identified in the promoter of the mouse *VDUP1* gene [59]. Furthermore, a clear induction in TXNIP/VDUP1 expression levels by 1,25(OH)_2_D_3_ has been largely limited to HL-60 cells [60]. Therefore, it is possible that the 1,25(OH)_2_D_3_ regulation of TXNIP, and subsequently glucose uptake, is subject to regulation of upstream pathways by treatment, and that the “canonical” induction in TXNIP expression by 1,25(OH)_2_D_3_ may not be observed in different tumor types. Further studies are needed to elucidate the effect of 1,25(OH)_2_D_3_ treatment on the interactions between these metabolic networks in cancer cells.

## 3. Vitamin D and Autophagy in Cancer: Friend or Foe?

Autophagy is a highly-conserved pathway used by cells to eliminate waste products and dysfunctional organelles [50,61]. It is a survival-promoting pathway that enables cells to overcome stressors such as nutrient deprivation, by degrading carbohydrates, proteins, and lipids to precursors that could be incorporated into energy-producing pathways [61]. With regards to cancer, autophagy has been shown to play roles in both oncogenesis and tumor suppression [50]. For example, autophagy deficiency has been connected to oxidative stress and genomic instability, known drivers of tumorigenesis [50]. On the other hand, the versatile metabolic precursors that autophagy activation produces can confer metabolic plasticity to cancer cells, which enables them to cope with stress imposed by the tumor microenvironment or therapy [61].

Emerging data have shown that autophagy is activated by 1,25(OH)_2_D_3_ and its analogues in different cell types [26,62,63]. This has been shown to be a route through which the molecule induces beneficial effects—for example, in the elimination of *Mycobacterium tuberculosis* through the induction of cathelicidin (an anti-microbial peptide) [64], and, in spite of the pathway’s controversial role in the disease, anti-tumor effects [62].

Different studies have reported a number of mechanisms through which VDR activators induce autophagy in breast cancer cells [26,65]. Høyer-Hansen et al. [26] demonstrated that the calcitriol analogue EB1089, among other Ca^2+^ mobilizing agents, induces autophagy in MCF-7 cells through activating the CAMKK2-AMPK pathway. Additionally, Tavera-Mendoza et al. [65] recently showed that in luminal breast cancer cells, 1,25(OH)_2_D_3_ induces an autophagic transcriptional signature that is found in normal mammary glands and lost during malignant transformation. The authors also demonstrated that the *MAP1LC31B* gene, which encodes the autophagic protein LC3B, harbors VDRE, and proposed that the autophagy associated with 1,25(OH)_2_D_3_ treatment is not pro-survival, since the molecule exerts clear anti-proliferative effects on the investigated models, as well as induces an anti-tumor gene expression landscape [65]. Furthermore, they showed that autophagosome accumulation resulting from the co-treatment of breast cancer cells with 1,25(OH)_2_D_3_ (an inducer of autophagosome formation) and chloroquine (an autophagosome acidification inhibitor) leads to the profound inhibition of proliferation in a manner more potent than that achieved by either molecule alone [65]. Similarly, we have recently shown that the combination of 1,25(OH)_2_D_3_ and metformin synergistically induces autophagy, assessed by the LC3II:LC3I ratio, as well as inhibits the proliferation of human colorectal cancer cells harboring wild-type p53 [66].

In view of these studies, it would be interesting to investigate whether the effects of VDR activators on autophagy are the result of the transcriptional regulation of autophagy genes, or secondary to the cellular metabolic events that occur upon treatment, or an interplay of both. Moreover, whether the effect of vitamin D compounds on autophagy induction is universal or dependent on genetic background and subject to the mutational landscape of the investigated tumor cells remains to be investigated in different cancer types. For example, in respect to p53, as detailed above, it has been shown that the p53 status influences the transcriptional activity of the VDR [35], as well as autophagy induction by 1,25(OH)_2_D_3_ [66]. Further investigations are required to elucidate the fate of cells that undergo autophagy in response to 1,25(OH)_2_D_3_ in different tumors, and the possible combination of VDR activators with pharmacological modulators of autophagy to enhance the molecules’ anti-cancer potential.

## 4. Concluding Remarks

Although profound progress has been made in understanding the complexity of vitamin D biology in transformed cells, the influence of calcitriol on the nutrient utilization of tumors is not clearly understood. 1,25(OH)_2_D_3_ appears to engage in complex, and at times paradoxical metabolic programs in cancer cells, such as the activation of AMPK and autophagic signaling, as well as the induction of G6PD and thus possibly the enhancement of the PPP. There also remain several uncharted territories regarding our understanding of calcitriol’s role in the regulation of cellular energy metabolism. For example, it is becoming increasingly clear that despite glutamine taking center stage in tumor cell specific amino acid metabolism, other essential and non-essential amino acids also play pivotal roles in sustaining cancer cell survival and proliferation [67]. Whether 1,25(OH)_2_D_3_ influences the utilization of these amino acids in tumor cells is yet to be thoroughly explored. We should also note that the influence of vitamin D on energy utilization may not exclusively be the result of the molecule’s effects on tumor cells, as some recent studies have shown that vitamin D may also induce tumor stroma reprogramming [68,69]. Thus, by modifying microenvironment conditions that are known to drive oncogenic metabolism, such as hypoxia [28], as well as by facilitating the entry of classical chemotherapeutics, such as gemcitabine and 5-Fluorouracil, also known to influence metabolism [7], vitamin D treatment could further impact nutrient metabolizing pathways in cancer cells. Furthermore, additional studies are needed to clarify whether calcitriol’s metabolism-modulating activities are primary effects or rather a secondary consequence of the calcitriol-mediated regulation of diverse tumor suppressors, oncogenes, and energy-/glucose-sensing signaling networks. Increasing our current understanding of how calcitriol and its analogues modulate these cancer hallmarks should enable the combination of these effects with established and newly designed chemotherapeutics, with the aim of achieving reciprocal synergy.

## Abbreviations

1,25(OH)_2_D_3_	1,25-dihydroxyvitamin D_3_
AMPK	AMP-activated protein kinase
CAMKK2	Calcium-calmodulin dependent kinase kinase 2
DDIT4	DNA damage inducible transcript 4
FASN	Fatty acid synthase
G6PD	Glucose-6-phosohate dehydrogenase
GLUT1	Glucose transporter 1
HIF1a	Hypoxia-inducible factor 1a
LC3	Light chain 3
LDHA	Lactate dehydrogenase A
LKB1	Liver kinase B1
mTOR	Mammalian target of rapamycin
mTORC1	Mammalian target of rapamycin compex 1
PDK	Pyruvate dehydrogenase kinase
PFK1	Phosphofructokinase 1
PKM2	Pyruvate kinase M2
PPP	Pentose phosphate pathway
Raptor	Regulatory-associated protein of mTOR
REDD1	Regulated in development and DNA damage response 1
Rheb	Ras homologue enriched in brain
TCA cycle	Tricarboxylic acid cycle
TIGAR	TP53-induced glycolysis and apoptosis regulator
TSC	Tuberous sclerosis complex
TXNIP	Thioredoxin-interacting protein
VDR	Vitamin D receptor
VDRE	Vitamin D response elements
VDUP1	Vitamin D_3_-upregulated protein 1

## References

[1] Vander Heiden M.G., Cantley L.C., Thompson C.B. (2009). Understanding the warburg effect: The metabolic requirements of cell proliferation. Science.

[2] Lopez-Lazaro M. (2008). The warburg effect: Why and how do cancer cells activate glycolysis in the presence of oxygen?. Anticancer Agents Med. Chem..

[3] Levine A.J., Puzio-Kuter A.M. (2010). The control of the metabolic switch in cancers by oncogenes and tumor suppressor genes. Science.

[4] Adekola K., Rosen S.T., Shanmugam M. (2012). Glucose transporters in cancer metabolism. Curr. Opin. Oncol..

[5] Christofk H.R., Vander Heiden M.G., Harris M.H., Ramanathan A., Gerszten R.E., Wei R., Fleming M.D., Schreiber S.L., Cantley L.C. (2008). The M2 splice isoform of pyruvate kinase is important for cancer metabolism and tumour growth. Nature.

[6] Miao P., Sheng S., Sun X., Liu J., Huang G. (2013). Lactate dehydrogenase a in cancer: A promising target for diagnosis and therapy. IUBMB Life.

[7] Vander Heiden M.G. (2011). Targeting cancer metabolism: A therapeutic window opens. Nat. Rev. Drug Discov..

[8] Shangary S., Wang S. (2009). Small-molecule inhibitors of the MDM2-p53 protein-protein interaction to reactivate p53 function: A novel approach for cancer therapy. Annu. Rev. Pharmacol. Toxicol..

[9] Yin X., Giap C., Lazo J.S., Prochownik E.V. (2003). Low molecular weight inhibitors of myc-max interaction and function. Oncogene.

[10] Wise D.R., Thompson C.B. (2010). Glutamine addiction: A new therapeutic target in cancer. Trends Biochem. Sci..

[11] Yang M., Vousden K.H. (2016). Serine and one-carbon metabolism in cancer. Nat. Rev. Cancer.

[12] Currie E., Schulze A., Zechner R., Walther T.C., Farese R.V. (2013). Cellular fatty acid metabolism and cancer. Cell Metab..

[13] Carracedo A., Cantley L.C., Pandolfi P.P. (2013). Cancer metabolism: Fatty acid oxidation in the limelight. Nat. Rev. Cancer.

[14] Zheng W., Tayyari F., Gowda G.A., Raftery D., McLamore E.S., Shi J., Porterfield D.M., Donkin S.S., Bequette B., Teegarden D. (2013). 1,25-dihydroxyvitamin D regulation of glucose metabolism in harvey-ras transformed MCF10A human breast epithelial cells. J. Steroid Biochem. Mol. Biol..

[15] Zhou X., Zheng W., Nagana Gowda G.A., Raftery D., Donkin S.S., Bequette B., Teegarden D. (2016). 1,25-dihydroxyvitamin D inhibits glutamine metabolism in harvey-ras transformed MCF10A human breast epithelial cell. J. Steroid Biochem. Mol. Biol..

[16] Wilmanski T., Buhman K., Donkin S.S., Burgess J.R., Teegarden D. (2017). 1α,25-dihydroxyvitamin D inhibits de novo fatty acid synthesis and lipid accumulation in metastatic breast cancer cells through down-regulation of pyruvate carboxylase. J. Nutr. Biochem..

[17] Abu El Maaty M.A., Alborzinia H., Khan S.J., Buttner M., Wolfl S. (2017). 1,25(OH)2D3 disrupts glucose metabolism in prostate cancer cells leading to a truncation of the TCA cycle and inhibition of txnip expression. Biochim. Biophys. Acta.

[18] Holick M.F. (2007). Vitamin D deficiency. N. Engl. J. Med..

[19] Feldman D., Krishnan A.V., Swami S., Giovannucci E., Feldman B.J. (2014). The role of vitamin D in reducing cancer risk and progression. Nat. Rev. Cancer.

[20] Deeb K.K., Trump D.L., Johnson C.S. (2007). Vitamin D signalling pathways in cancer: Potential for anticancer therapeutics. Nat. Rev. Cancer.

[21] Abu El Maaty M.A., Wolfl S. (2017). Effects of 1,25(OH)(2)D(3) on cancer cells and potential applications in combination with established and putative anti-cancer agents. Nutrients.

[22] Reichrath J., Reichrath S., Heyne K., Vogt T., Roemer K. (2014). Tumor suppression in skin and other tissues via cross-talk between vitamin D- and p53-signaling. Front. Physiol..

[23] Dang C.V. (2013). Myc, metabolism, cell growth, and tumorigenesis. Cold Spring Harb. Perspect. Med..

[24] Semenza G.L. (2010). Hif-1: Upstream and downstream of cancer metabolism. Curr. Opin. Genet. Dev..

[25] Berkers C.R., Maddocks O.D., Cheung E.C., Mor I., Vousden K.H. (2013). Metabolic regulation by p53 family members. Cell Metab..

[26] Hoyer-Hansen M., Bastholm L., Szyniarowski P., Campanella M., Szabadkai G., Farkas T., Bianchi K., Fehrenbacher N., Elling F., Rizzuto R. (2007). Control of macroautophagy by calcium, calmodulin-dependent kinase kinase-beta, and Bcl-2. Mol. Cell.

[27] Lisse T.S., Liu T., Irmler M., Beckers J., Chen H., Adams J.S., Hewison M. (2011). Gene targeting by the vitamin D response element binding protein reveals a role for vitamin D in osteoblast mtor signaling. FASEB J..

[28] Gordan J.D., Thompson C.B., Simon M.C. (2007). Hif and c-myc: Sibling rivals for control of cancer cell metabolism and proliferation. Cancer Cell.

[29] Ben-Shoshan M., Amir S., Dang D.T., Dang L.H., Weisman Y., Mabjeesh N.J. (2007). 1α,25-dihydroxyvitamin D3 (calcitriol) inhibits hypoxia-inducible factor-1/vascular endothelial growth factor pathway in human cancer cells. Mol. Cancer Ther..

[30] Polek T.C., Stewart L.V., Ryu E.J., Cohen M.B., Allegretto E.A., Weigel N.L. (2003). P53 is required for 1,25-dihydroxyvitamin D3-induced G0 arrest but is not required for G1 accumulation or apoptosis of LNCaP prostate cancer cells. Endocrinology.

[31] Salehi-Tabar R., Nguyen-Yamamoto L., Tavera-Mendoza L.E., Quail T., Dimitrov V., An B.S., Glass L., Goltzman D., White J.H. (2012). Vitamin D receptor as a master regulator of the C-MYC/MXD1 network. Proc. Natl. Acad. Sci. USA.

[32] Toropainen S., Vaisanen S., Heikkinen S., Carlberg C. (2010). The down-regulation of the human MYC gene by the nuclear hormone 1α,25-dihydroxyvitamin D3 is associated with cycling of corepressors and histone deacetylases. J. Mol. Biol..

[33] Wang T.T., Tavera-Mendoza L.E., Laperriere D., Libby E., MacLeod N.B., Nagai Y., Bourdeau V., Konstorum A., Lallemant B., Zhang R. (2005). Large-scale in silico and microarray-based identification of direct 1,25-dihydroxyvitamin D3 target genes. Mol. Endocrinol..

[34] Muller P.A., Vousden K.H. (2014). Mutant p53 in cancer: New functions and therapeutic opportunities. Cancer Cell.

[35] Stambolsky P., Tabach Y., Fontemaggi G., Weisz L., Maor-Aloni R., Siegfried Z., Shiff I., Kogan I., Shay M., Kalo E. (2010). Modulation of the vitamin D3 response by cancer-associated mutant p53. Cancer Cell.

[36] Tsui K.H., Feng T.H., Lin Y.F., Chang P.L., Juang H.H. (2011). P53 downregulates the gene expression of mitochondrial aconitase in human prostate carcinoma cells. Prostate.

[37] Ferreira G.B., Vanherwegen A.S., Eelen G., Gutierrez A.C., Van Lommel L., Marchal K., Verlinden L., Verstuyf A., Nogueira T., Georgiadou M. (2015). Vitamin D3 induces tolerance in human dendritic cells by activation of intracellular metabolic pathways. Cell Rep..

[38] Ryan Z.C., Craig T.A., Folmes C.D., Wang X., Lanza I.R., Schaible N.S., Salisbury J.L., Nair K.S., Terzic A., Sieck G.C. (2016). 1α,25-dihydroxyvitamin D3 regulates mitochondrial oxygen consumption and dynamics in human skeletal muscle cells. J. Biol. Chem..

[39] Contractor T., Harris C.R. (2012). P53 negatively regulates transcription of the pyruvate dehydrogenase kinase PDK2. Cancer Res..

[40] Stanton R.C. (2012). Glucose-6-phosphate dehydrogenase, nadph, and cell survival. IUBMB Life.

[41] Noun A., Garabedian M., Monet J.D. (1989). Stimulatory effect of 1,25-dihydroxyvitamin D3 on the glucose-6-phosphate dehydrogenase activity in the MCF-7 human breast cancer cell line. Cell Biochem. Funct..

[42] Simmons K.M., Beaudin S.G., Narvaez C.J., Welsh J. (2015). Gene signatures of 1,25-dihydroxyvitamin D3 exposure in normal and transformed mammary cells. J. Cell. Biochem..

[43] Bao B.Y., Ting H.J., Hsu J.W., Lee Y.F. (2008). Protective role of 1α, 25-dihydroxyvitamin D3 against oxidative stress in nonmalignant human prostate epithelial cells. Int. J. Cancer.

[44] Patra K.C., Hay N. (2014). The pentose phosphate pathway and cancer. Trends Biochem. Sci..

[45] Swami S., Krishnan A.V., Feldman D. (2000). 1α,25-dihydroxyvitamin D3 down-regulates estrogen receptor abundance and suppresses estrogen actions in MCF-7 human breast cancer cells. Clin. Cancer Res..

[46] Simboli-Campbell M., Narvaez C.J., Tenniswood M., Welsh J. (1996). 1,25-dihydroxyvitamin D3 induces morphological and biochemical markers of apoptosis in MCF-7 breast cancer cells. J. Steroid Biochem. Mol. Biol..

[47] Hardie D.G., Ross F.A., Hawley S.A. (2012). Amp-activated protein kinase: A target for drugs both ancient and modern. Chem. Biol..

[48] Liang J., Mills G.B. (2013). AMPK: A contextual oncogene or tumor suppressor?. Cancer Res..

[49] Park H.U., Suy S., Danner M., Dailey V., Zhang Y., Li H., Hyduke D.R., Collins B.T., Gagnon G., Kallakury B. (2009). AMP-activated protein kinase promotes human prostate cancer cell growth and survival. Mol. Cancer Ther..

[50] White E. (2015). The role for autophagy in cancer. J. Clin. Investig..

[51] Laplante M., Sabatini D.M. (2009). Mtor signaling at a glance. J. Cell Sci..

[52] Wu N., Zheng B., Shaywitz A., Dagon Y., Tower C., Bellinger G., Shen C.H., Wen J., Asara J., McGraw T.E. (2013). AMPK-dependent degradation of txnip upon energy stress leads to enhanced glucose uptake via GLUT1. Mol. Cell.

[53] Chen K.S., DeLuca H.F. (1994). Isolation and characterization of a novel cdna from HL-60 cells treated with 1,25-dihydroxyvitamin D-3. Biochim. Biophys. Acta.

[54] Nishiyama A., Matsui M., Iwata S., Hirota K., Masutani H., Nakamura H., Takagi Y., Sono H., Gon Y., Yodoi J. (1999). Identification of thioredoxin-binding protein-2/vitamin D(3) up-regulated protein 1 as a negative regulator of thioredoxin function and expression. J. Biol. Chem..

[55] Stoltzman C.A., Peterson C.W., Breen K.T., Muoio D.M., Billin A.N., Ayer D.E. (2008). Glucose sensing by mondoa:Mlx complexes: A role for hexokinases and direct regulation of thioredoxin-interacting protein expression. Proc. Natl. Acad. Sci. USA.

[56] Kaadige M.R., Yang J., Wilde B.R., Ayer D.E. (2015). Mondoa-MLX transcriptional activity is limited by mtor-mondoa interaction. Mol. Cell. Biol..

[57] Jin H.O., Seo S.K., Kim Y.S., Woo S.H., Lee K.H., Yi J.Y., Lee S.J., Choe T.B., Lee J.H., An S. (2011). Txnip potentiates REDD1-induced mtor suppression through stabilization of REDD1. Oncogene.

[58] Zhou J., Yu Q., Chng W.J. (2011). Txnip (VDUP-1, TBP-2): A major redox regulator commonly suppressed in cancer by epigenetic mechanisms. Int. J. Biochem. Cell Biol..

[59] Ludwig D.L., Kotanides H., Le T., Chavkin D., Bohlen P., Witte L. (2001). Cloning, genetic characterization, and chromosomal mapping of the mouse VDUP1 gene. Gene.

[60] Shalev A. (2014). Minireview: Thioredoxin-interacting protein: Regulation and function in the pancreatic beta-cell. Mol. Endocrinol..

[61] Kimmelman A.C., White E. (2017). Autophagy and tumor metabolism. Cell Metab..

[62] Wang J., Lian H., Zhao Y., Kauss M.A., Spindel S. (2008). Vitamin D3 induces autophagy of human myeloid leukemia cells. J. Biol. Chem..

[63] Wang R.C., Levine B. (2011). Calcipotriol induces autophagy in hela cells and keratinocytes. J. Investig. Dermatol..

[64] Yuk J.M., Shin D.M., Lee H.M., Yang C.S., Jin H.S., Kim K.K., Lee Z.W., Lee S.H., Kim J.M., Jo E.K. (2009). Vitamin D3 induces autophagy in human monocytes/macrophages via cathelicidin. Cell Host Microbe.

[65] Tavera-Mendoza L.E., Westerling T., Libby E., Marusyk A., Cato L., Cassani R., Cameron L.A., Ficarro S.B., Marto J.A., Klawitter J. (2017). Vitamin D receptor regulates autophagy in the normal mammary gland and in luminal breast cancer cells. Proc. Natl. Acad. Sci. USA.

[66] Abu El Maaty M.A., Strassburger W., Qaiser T., Dabiri Y., Wolfl S. (2017). Differences in p53 status significantly influence the cellular response and cell survival to 1,25-dihydroxyvitamin D3-metformin cotreatment in colorectal cancer cells. Mol. Carcinog..

[67] Lukey M.J., Katt W.P., Cerione R.A. (2017). Targeting amino acid metabolism for cancer therapy. Drug Discov. Today.

[68] Sherman M.H., Yu R.T., Engle D.D., Ding N., Atkins A.R., Tiriac H., Collisson E.A., Connor F., Van Dyke T., Kozlov S. (2014). Vitamin D receptor-mediated stromal reprogramming suppresses pancreatitis and enhances pancreatic cancer therapy. Cell.

[69] Ferrer-Mayorga G., Gomez-Lopez G., Barbachano A., Fernandez-Barral A., Pena C., Pisano D.G., Cantero R., Rojo F., Munoz A., Larriba M.J. (2016). Vitamin D receptor expression and associated gene signature in tumour stromal fibroblasts predict clinical outcome in colorectal cancer. Gut.

